# Green Chemistry: Wilson and Schwarzman Respond

**DOI:** 10.1289/ehp.0900835R

**Published:** 2009-09

**Authors:** Michael P. Wilson, Megan R. Schwarzman

**Affiliations:** University of California, Berkeley, California, E-mail: mpwilson@berkeley.edu

We commend O’Brien, Myers, and Warner for their pioneering work identifying chemical threats to human and ecosystem health and for advancing the field of green chemistry. In our article (Wilson and Schwarzman 2009), we discussed advancing green chemistry as an objective of chemicals policy and described existing policy barriers that, if lifted, would spur the scientific and commercial development of green chemistry. O’Brien et al. recognize that the science of green chemistry has a role in informing chemicals policies. We would add that public policy that accurately reflects current science—and the needs of the chemicals market—is instrumental to the widespread adoption of green chemistry, but dispute that green chemistry is integral to chemicals policy itself.

Green chemistry’s potential is expanded when the science is embedded in a larger context. For example, in an introductory text on green chemistry, Lancaster (2002) stated that

Green chemistry is not a new branch of science, but more a new philosophical approach that underpins all of chemistry and has technological, environmental and societal goals.

Lancaster (2002) pointed out that “over the last ten years, green chemistry has gradually become recognized as both a culture and a methodology for achieving sustainability,” and that the “12 principles of green chemistry help show how this can be achieved” (Lancaster 2002). This book addresses technical aspects of green chemistry while acknowledging the economic, legal, and knowledge barriers to advancing green chemistry.

California has adopted a similarly expansive approach. The state’s 2-year old Green Chemistry Initiative is rooted in the 12 principles of green chemistry, and it also embraces a range of tools to ensure the success of green chemistry in society. These include new regulatory strategies, economic incentives, technical assistance, research, and education, with the goal of “launch[ing] a new chemicals framework and a quantum shift in environmental protection” (Adams 2008). These are the same kinds of legislative and regulatory tools that California has used successfully to promote innovation in the energy sector, and which the empirical evidence has identified are the primary drivers for the adoption of cleaner technologies (Nameroff 2004). It is in this context that we see the broad potential of green chemistry.

It is a credit to those who have established the field of green chemistry that multiple interests recognize its value in achieving environmental and economic sustainability, with benefits for worker health, resource conservation, environmental justice, public health, and global warming. As a result, green chemistry is reaching out from a core set of technical principles to inform broad societal goals.

As the field of green chemistry garners increasing attention in the scientific community and in society, it is worth recognizing that the 12 principles of green chemistry will be put to use in many contexts. We welcome the opportunity to join others in engaging with this promising field, with its relationship to the environmental health sciences and its role in effective public policy.
