# Errata

**Published:** 2009-10

In the letter by Wilson and Schwarzman [Environ Health Perspect 117:A386 (2009)], the last sentence in the first paragraph was incorrect. The corrected sentence is as follows:

We would add that public policy that accurately reflects current science—and the needs of the chemicals market—is instrumental to the widespread adoption of green chemistry.

*EHP* apologizes for the error.

